# Knot spectrum of turbulence

**DOI:** 10.1038/s41598-019-47103-w

**Published:** 2019-07-22

**Authors:** R. G. Cooper, M. Mesgarnezhad, A. W. Baggaley, C. F. Barenghi

**Affiliations:** 1 School of Mathematics, Statistics and Physics Newcastle University, Newcastle upon Tyne, NE1 7RU UK; 2 JQC (Joint Quantum Centre), Durham-Newcastle, UK

**Keywords:** Condensed-matter physics, Fluid dynamics

## Abstract

Streamlines, vortex lines and magnetic flux tubes in turbulent fluids and plasmas display a great amount of coiling, twisting and linking, raising the question as to whether their topological complexity (continually created and destroyed by reconnections) can be quantified. In superfluid helium, the discrete (quantized) nature of vorticity can be exploited to associate to each vortex loop a knot invariant called the Alexander polynomial whose degree characterizes the topology of that vortex loop. By numerically simulating the dynamics of a tangle of quantum vortex lines, we find that this quantum turbulence always contains vortex knots of very large degree which keep forming, vanishing and reforming, creating a distribution of topologies which we quantify in terms of a knot spectrum and its scaling law. We also find results analogous to those in the wider literature, demonstrating that the knotting probability of the vortex tangle grows with the vortex length, as for macromolecules, and saturates above a characteristic length, as found for tumbled strings.

## Introduction

## Motivation

Tangled filamentary structures are a regular occurrence in physical systems ranging from turbulent fluids^1^ and magnetic fields^2,3^ to optics^4,5^, nematic liquid crystals^6^ and superfluids^7^. Knowing if such structures are knotted is important; for example, in DNA this knowledge provides valuable information on the global arrangement of molecules^8^. In fluids, plasmas and superfluids any increase or decrease of linking or knottiness is caused by vortex reconnections^9–11^, dramatic events which are associated with viscous, resistive and acoustic energy losses respectively. One would like to relate the topology of turbulent flows to their energy, and the first step in this direction is to precisely quantify the topology. The success in creating a single vortex knot in controlled laboratory conditions^12^ has stimulated new ideas, including the use of polynomials to analyse knotted structures. For example, it has been shown that the decay of a single knotted vortex^13^ can be tracked using the HOMFLYPT polynomial^14^. Unfortunately, the step from one vortex to turbulence is still a big step. In computational fluid dynamics, a sample of vortex lines in a turbulent flow can be traced and analyzed (at least in principle) to determine whether they are knotted or linked; however, moving across regions of high and low background vorticity, we may find that, due to unavoidable numerical noise, the line does not close as required by the solenoidal property of vorticity.

To overcome this difficulty we note that whereas in ordinary fluids vorticity is a continuous field of arbitrary shape and strength, in quantum fluids^15^ such as superfluid helium and atomic Bose-Einstein condensates (BECs), vorticity consists of individual vortex lines of fixed circulation $\kappa =h/m$ (where *h* is Planck’s constant and *m* the mass of the relevant boson) moving in the perfect background of inviscid potential flow. In these quantum systems, a vortex line is an individual topological defect (the phase of the complex wave function ${\rm{\Psi }}$ vanishes on the vortex axis, hence its phase is undefined) around which the phase changes by 2*π*, corresponding to the superflow $v=\kappa /(2\pi r)$ where *r* is the radial distance to the axis. This property guarantees that (away from boundaries where $|{\rm{\Psi }}|\to 0$) there is never any numerical ambiguity in localizing a vortex line, hence determining the linking and the knottiness of lines. It is therefore convenient to study the problem of the topology of turbulence in the context of turbulent quantum fluids. This state of ‘quantum turbulence’ can be easily generated in the laboratory by stirring liquid helium or atomic condensates, and takes the form of a disordered tangle of vortex lines.

An additional motivation to consider the topology of quantum turbulence is that if the superfluid is forced at length scales *D* much larger than the average distance $\ell$ between vortex lines^16–18^ so that enough k-space is available, then, in this wide range between *D* and $\ell$ the quantum turbulent flow displays the same velocity statistics^19,20^ and the same^21^ power-law distribution of kinetic energy (the Kolmogorov law) which is observed in ordinary (classical) turbulence. In this particular regime, quantum turbulence therefore represents the ‘skeleton’ of classical turbulence (other regimes of quantum turbulence exist which do not display the Kolmogorov law and lack an energy cascade^18^).

Thus motivated, our aim is to explore numerically the topology of quantum turbulence. At this early stage of investigation, the precise state of quantum turbulence which is examined must be chosen on the ground of its simplicity and experimental availability.

As in classical fluids, a quantum vortex line is either a closed loops or it terminates at a boundary. To avoid any ambiguity or arbitrariety in characterizing the linking between two vortex lines, we restrict our attention to turbulence in an infinite domain, away from boundaries (assuming zero velocity at infinity), thus guaranteeing that all vortex lines are in the shape of closed loops. A further simplification is to restrict our study to statistical steady state regimes, such that forcing and dissipation are balanced, flow properties fluctuate around mean values, and the memory of the initial condition is lost.

These considerations lead us to consider experiments in which quantum turbulence is generated at the centre of the experimental cell away from boundaries at sufficiently high temperatures that the mutual friction between the vortex lines and the thermal excitations (the normal fluid) provides a mechanism to inject (extract) energy into (from) the vortex lines; this temperature regime is also the most common in experiments. To sustain turbulence at the centre of the sample, the liquid helium must be stirred by suitably positioned small oscillating objects^22^, such as forks^23^, grids^24,25^, or wires^26^, or by focusing ultrasound^27,28^; in atomic BECs, the analog set-up would be to create a localized tangle of vortex lines by stirring a large condensate with a laser spoon.

**Figure 1:**
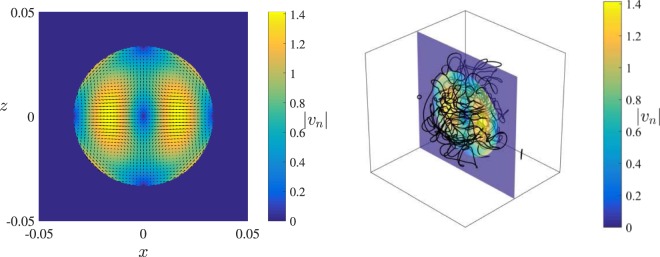
Left: Instantaneous Dudley-James flow: plot of $|{{\bf{v}}}_{n}|$ vs *x*, *z* at $y=0$ in the region $-0.05 < x$, $y < 0.05\,{\rm{cm}}$ with superimposed arrowplots. Right: Instantaneous three-dimensional snapshot of the vortex tangle superimposed to the magnitude $|{{\bf{v}}}_{n}|$ of the driving Dudley-James flow on the *x*, *z* plane at $y=0$. The cube $-0.05 < x$, *y*, $z < 0.05\,{\rm{cm}}$ around the vortex tangle is for visualization only (the simulation is performed in an infinite domain).

Since we are not interested in modelling the detailed action of a specific oscillating object on the liquid helium but only in feeding/extracting energy into/from the vortex tangle in a localised region, we model the normal fluid’s velocity field using a relatively simple, localised, time-dependent flow. Such flow is usually laminar (as Reynolds numbers based on the viscosity of the normal fluid and the velocity/size of small oscillating objects are never large). We choose a Dudley-James flow^29^ within a spherical region of radius $D=0.03\,{\rm{cm}}$ and zero outside (see Fig. 1(left) and Methods for details). Unlike the random waves used in a preliminary investigation^30^ which focused on the superfluid helicity, the Dudley-James flow is incompressible, hence more realistic. Dudley-James flows have been used in MHD to study the kinematic dynamo (the growth and nonlinear saturation of magnetic field given a prescribed velocity field), a problem which is (at least in spirit) similar to ours (the growth and nonlinear saturation of a tangle of quantum vortex lines given a prescribed normal fluid velocity field). The initial condition of each numerical simulation consists of a few random seeding vortex rings. The evolution of this initial condition into a turbulent tangle of vortex lines in a statistical steady-state is simulated using the Vortex Filament Method (see Methods for details).

## Results

At the beginning of the time evolution, the length of the initial vortex configuration increases rapidly as energy is fed into the vortices by the normal fluid via the Donnelly-Glaberson (DG) instability^31^. The DG instability manifests itself as growing Kelvin waves - helical displacements of the vortex lines away from their initial position - and occurs if the component of the normal fluid velocity parallel to a vortex line exceeds a critical value. Closely packed vortex lines interact, stretching and rotating around each other, and reconnect when they collide^32,33^. The relaxation of each reconnection cusp releases more Kelvin waves^34^, and a turbulent tangle of vortex lines is quickly created.

**Figure 2:**
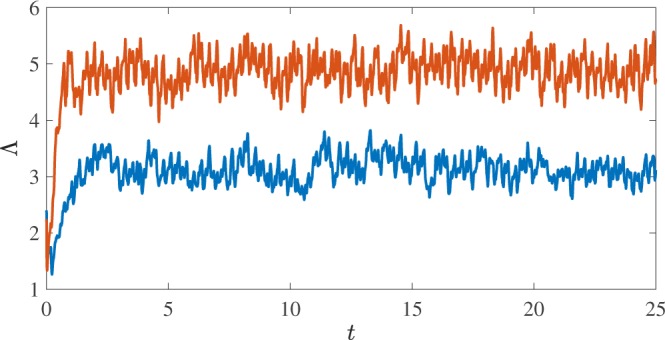
Time evolution of the vortex line length ${\rm{\Lambda }}$ (cm) in the two numerical simulations. The lower/upper (blue/red) lines correspond to normal fluid drives of ${v}_{f}=4.75\,{\rm{cm}}/{\rm{s}}$ and ${v}_{f}=5.25\,{\rm{cm}}/{\rm{s}}$ respectively.

After the initial transient, the growth of the vortex line length is balanced by dissipation and the vortex tangle settles to a statistical steady-state in which the total vortex length fluctuates around a mean value which depends on the driving normal fluid velocity, as shown in Fig. 2. This happens, firstly because the friction with the normal fluid damps out the Kelvin waves^35^; secondly because the vortex loops which escape the central region (where the normal fluid stirs the turbulence) shrink and vanish due to the friction with the stationary normal fluid in the outer region (no DG instability takes place there). The vortex tangle, therefore, remains localised in the region of the driving normal flow, as shown in Fig. 1(right).

**Figure 3:**
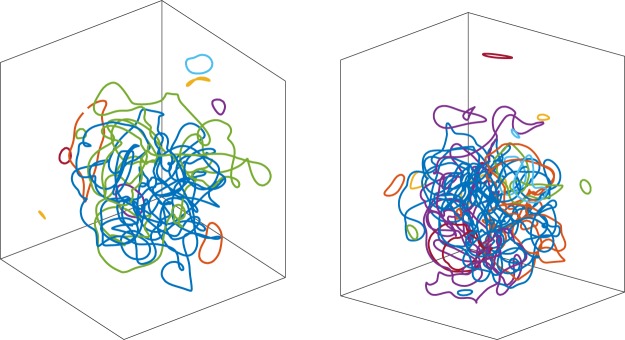
Snapshots of the vortex configuration for the lower (left) and higher (right) normal fluid drives ${v}_{f}=4.75\,{\rm{cm}}/{\rm{s}}$ and ${v}_{f}=5.25\,{\rm{cm}}/{\rm{s}}$ respectively at *t* = 20 s in the saturated steady-state regime. Different colours are used to identify distinct vortex lines in the two snapshots. The cubes around the vortex tangles are for visualization only (the simulations are performed in an infinite domain).

Snapshots of the vortex configurations for the two different levels of normal fluid drive are plotted in Fig. 3: the larger the driving normal fluid velocity, the more intense the turbulence (its intensity is traditionally quantified in experiments in terms of the vortex line density *L*, the length of vortex line per unit volume). At temperatures lower than the results presented here, the turbulent tangles shown in Fig. 3 would be surrounded by a cloud of small vortex loops which rapidly fly away^36^; at the temperatures which we consider here these small loops are destroyed by friction. In either case, by either shrinking and vanishing (at intermediate and high temperatures) or by rapidly flying away (at low temperatures) these vortex loops represent an energy sink at short length scales. In this way a balance is reached between drive and dissipation, resulting in fluctuations of the vortex length and the energy about mean values.

**Figure 4:**
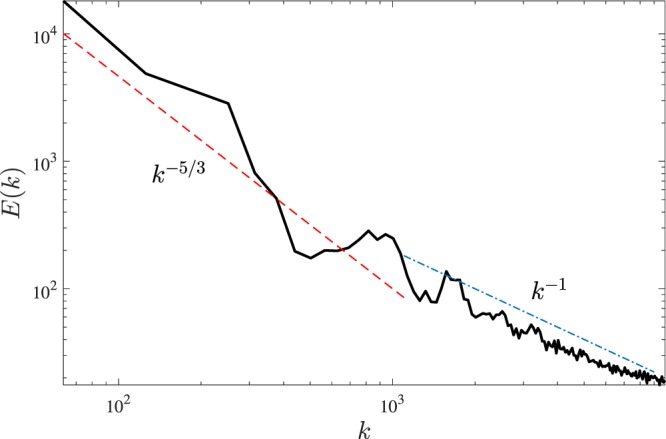
Instantaneous energy spectrum *E*(*k*) (arbitrary units) of the superfluid velocity at $t=24\,{\rm{s}}$ (in the saturated regime) for ${v}_{f}=5.25\,{\rm{cm}}/{\rm{s}}$ drive plotted vs wavenumber *k* (cm^−1^). The red and blue dashed lines are guides to the eye to indicate the *k*^−5/3^ and the *k*^−1^ scaling slopes respectively. The crossover between the two behaviours corresponds to the average intervortex distance $\ell$.

To demonstrate in a quantitative way that our vortex configurations are turbulent, Fig. 4 shows the kinetic energy spectrum *E*(*k*) (where *k* is the magnitude of the three-dimensional wavenumber **k**) in the saturated regime. It is apparent that the energy injected at the large length scale *D* cascades to smaller length scales (larger wavenumbers *k*). As the total injected energy is balanced by friction, most energy remains contained in the length scales larger than the average intervortex distance $\ell$. In this region ${k}_{D} < k < {k}_{\ell }$ of k-space (where ${k}_{D}=2\pi$/*D* and ${k}_{\ell }=2\pi$/$\ell$), we find that the energy spectrum is consistent with the famous Kolmogorov scaling $E(k)\sim {k}^{-5/3}$ of classical homogeneous isotropic turbulence. We stress that we should not expect a precise *k*^−5/3^ scaling in our simulations: our turbulent flow is not homogeneous and only one decade of k-space is available. The crossover from the Kolmogorov *k*^−5/3^ scaling to the $E(k)\sim {k}^{-1}$ scaling which is typical of individual vortex lines occurs at approximately $k\approx {10}^{3}\,{{\rm{cm}}}^{-1}$, which is consistent with ${k}_{\ell }\approx 1300\,{{\rm{cm}}}^{-1}$ estimated from the vortex line density $L={\rm{\Lambda }}$/*V* where $V\approx 4\pi {D}^{3}$/3 is the volume of the turbulent region and $\ell \approx {L}^{-1/2}\approx 4.8\times {10}^{-3}\,{\rm{cm}}$ is the average intervortex spacing corresponding to the numerical simulation at large drive (${\rm{\Lambda }}\approx 5\,{\rm{cm}}$).

Our technique to quantify the topology of the turbulence is based on a knot invariant: the Alexander polynomial^37^. The polynomial ${\rm{\Delta }}(\tau )={a}_{0}+{a}_{1}\tau +\cdots +{a}_{\nu }{\tau }^{\nu }$ of degree $\nu$ with integer coefficients ${a}_{0},\ldots ,{a}_{\nu }$ is assigned to a knot type. For example, a vortex ring has Alexander polynomial ${\rm{\Delta }}(\tau )=1$ which identifies the *unknot*; therefore any closed vortex loop which can be continuously (i.e. without reconnections) deformed into a ring corresponds to ${\rm{\Delta }}(\tau )=1$. The simplest non-trivial knot is the *trefoil knot* which has Alexander polynomial ${\rm{\Delta }}(\tau )=1-\tau +{\tau }^{2}$ of degree $\nu =2$. In general, the higher the degree $\nu$ of the Alexander polynomial, the more complex the knot type of the vortex loop.

The instantaneous vortex configuration consists of *N* vortex loops ${ {\mathcal L} }_{i}$ ($i=1,\ldots N$), where *N* changes with time as vortex reconnections continually merge and split vortex loops. After the initial transient, *N* fluctuates about a mean value. In this statistical steady state regime, at any given time *t*, we numerically determine the Alexander polynomial of each loop ${ {\mathcal L} }_{i}$ ($i=1,\ldots N$) and call ${\nu }_{i}$ its degree. We stress that our simulations are not stochastic: the only source of randomness in the tangle’s topology arises from the turbulent fluctuations. Data are collected over time (in the steady-state regime) and cumulated to generate statistics for the analysis.

Our initial focus is what determines the probability that a given loop is knotted. Perhaps unsurprisingly, this fundamental question has been addressed in studies of other physical systems that contain knots. For example, Arsuaga *et al*.^38^ performed Monte Carlo simulations of random knotting in confined volumes, which were directly compared to the visualisation of DNA molecules. Their simulations suggested a linear relationship between the length of a loop and its probability of being knotted, which we shall denote *P*_*k*_. A more recent study by Raymer & Smith^39^ performed experiments where strings of different lengths were tumbled inside a box. They found that *P*_*k*_ grows rapidly with the length of the string, and saturates to some limiting value above a characteristic string length. Interestingly, the limiting value which they found was substantially less than 1, which they attributed to the finite agitation time and the stiffness of the strings.

**Figure 5:**
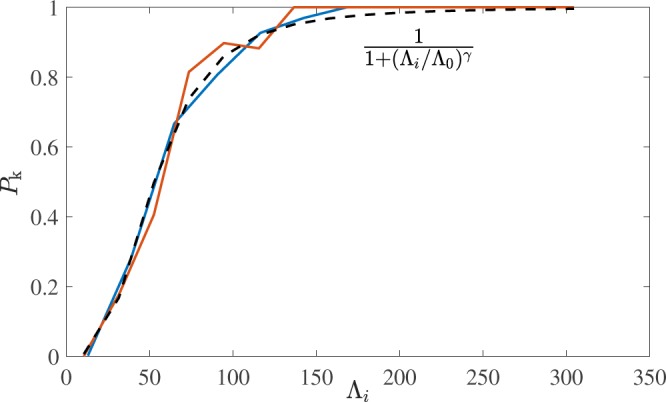
Probability *P*_*k*_ that a vortex loop is knotted plotted vs the loop’s length ${\rm{\Lambda }}$. We have fitted data from both simulations (${v}_{f}=4.75\,{\rm{cm}}/{\rm{s}}$ and $5.25\,{\rm{cm}}/{\rm{s}}$, red and blue curves respectively) with the same sigmoidal curve (black dashed curve) with fitting parameters ${{\rm{\Lambda }}}_{0}=53\,{\rm{cm}}$, $\gamma =-\,3.1$. Bin widths are taken to be approximately 20 cm.

**Figure 6:**
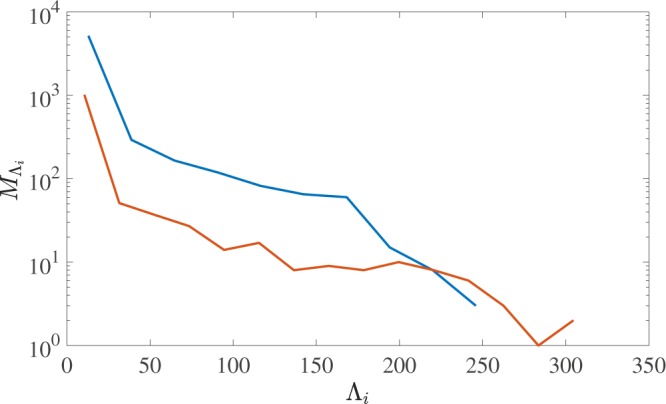
The number of knots, ${M}_{{{\rm{\Lambda }}}_{i}}$ within each of the bin widths, ${{\rm{\Lambda }}}_{i}$, used in Fig. 5.

We now proceed with the analysis of our results. Figure 5 displays ${P}_{k}({\rm{\Lambda }})$, the probability that a loop of length ${\rm{\Lambda }}$ is knotted, calculated in the saturated steady-state regimes for both small and large normal fluid drives considered in this study. We find that ${P}_{k}({\rm{\Lambda }})$ is independent of the normal fluid stirring (at least for the values considered here) and depends only on the loop length. Following Raymer & Smith^39^, we fit a sigmoidal curve to the data of the form ${P}_{k}({\rm{\Lambda }})=(1+{({\rm{\Lambda }}/{{\rm{\Lambda }}}_{0})}^{\gamma })$. Fitting our data using ${v}_{f}=4.75\,{\rm{cm}}/{\rm{s}}$, we find ${{\rm{\Lambda }}}_{0}=53\,{\rm{cm}}$ and $\gamma =-\,3.1$, in fair agreement with $\gamma =-\,2.9$ reported by Raymer & Smith^39^, suggesting that the knotting probabilities of quantised vortices moving under reconnecting Biot-Savart dynamics and tumbled strings are not unrelated. To show that our results are statistically robust in the limit of large ${{\rm{\Lambda }}}_{i}$, Fig. 6 displays the number of knots, ${M}_{{{\rm{\Lambda }}}_{i}}$, within each of the bins of the histogram. Notice that in general there are at least 10 vortex loops within a bin, until ${{\rm{\Lambda }}}_{i} > 250$ for the higher drive simulation which is well into the asymptotic regime of ${P}_{k}=1$.

**Figure 7:**
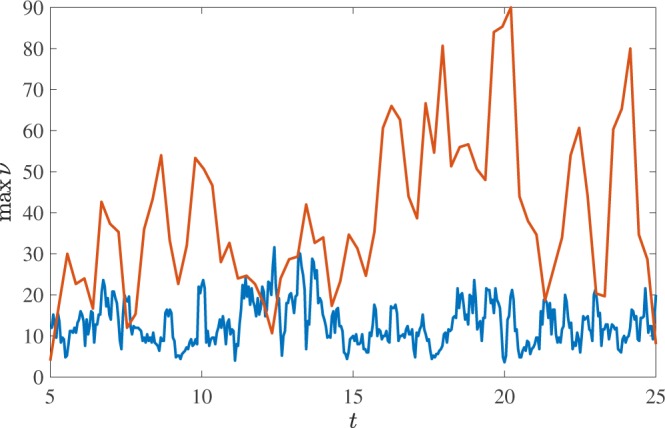
Time series of the largest degree of Alexander polynomial in the vortex configuration. The upper line is for ${v}_{f}=4.75\,{\rm{cm}}/{\rm{s}}$, and the lower line for ${v}_{f}=5.25\,{\rm{cm}}/{\rm{s}}$.

**Figure 8:**
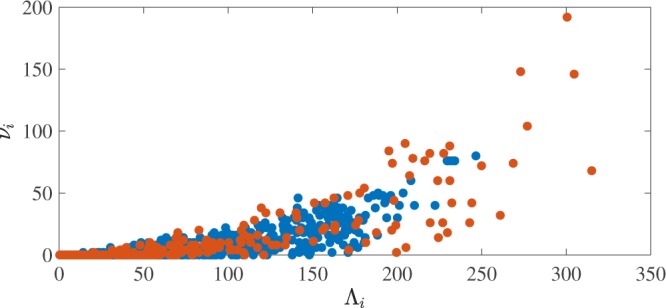
The degree ${\nu }_{i}$ of the Alexander polynomial of each vortex loop plotted against its length ${{\rm{\Lambda }}}_{i}$ (in cm) for the lower normal fluid drive (${v}_{f}=4.75\,{\rm{cm}}/{\rm{s}}$, blue symbols) and the higher drive (${v}_{f}=5.15\,{\rm{cm}}/{\rm{s}}$, red symbols).

We now move to consider the topological complexity of individual vortex loops and how it depends on the normal fluid drive and the loop’s length. As we have discussed, at any instant of time, the vortex tangle contains knots, and the most complex knot has the largest value of $\nu$. Figure 7 shows the time series of the maximal degree of Alexander polynomial present in the vortex tangle, revealing that at all times the vortex tangle contains a significant topological complexity - we never observe that the tangle is simply a collection of unknots. It must be stressed that the very knotted vortex structures which we detect are unstable: they are continually broken down by vortex reconnections, but they keep reforming by further reconnections. The natural question is whether the complexity depends on the driving normal fluid velocity. To answer the question we show a scatter-plot of ${{\rm{\Lambda }}}_{i}$ vs ${\nu }_{i}$, see Fig. 8. It is apparent that complexity is strongly related to the loop’s length, which is perhaps unsurprising. However, as for ${P}_{k}({\rm{\Lambda }})$, the underlying functional relationship between ${\nu }_{i}$ and ${{\rm{\Lambda }}}_{i}$ appears independent of *v*_*f*_, so the additional complexity which is evident in Fig. 8 is simply due to a stronger drive’s propensity to generate longer loops (larger values of ${{\rm{\Lambda }}}_{i}$).

**Figure 9:**
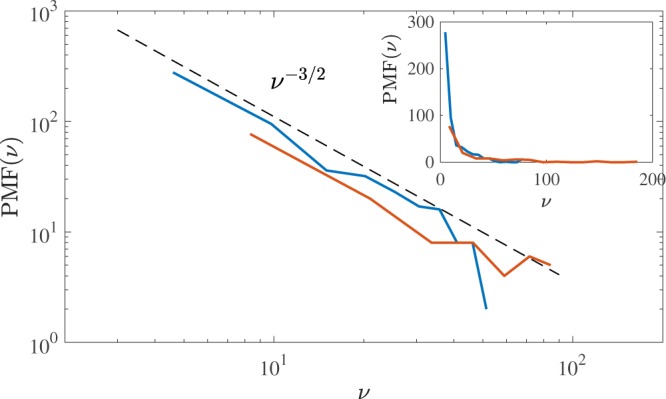
Probability mass functions (PMFs) of the degree of the Alexander polynomial $\nu$ plotted on a log-log scale (main graph) and linear-linear scale (inset). The logarithmic scale suggests $PMF(\nu )\sim {\nu }^{-3/2}$, as shown by the black dashed line.

Finally, attempting to gain a probabilistic understanding of the topological complexity of quantum turbulence which in the future could be compared to the distribution of knots in DNA, polymers, etc, we consider the distribution of ${\nu }_{i} > 0$. Figure 9 shows the Probability Mass Function (PMF) (normalized histogram of discrete variable) of the degrees of Alexander polynomials: ${\rm{PMF}}({\nu }_{i})$ vs ${\nu }_{i}$. We find two striking results: firstly that the PMFs are again independent of the normal fluid drive, and secondly that there appears a distinct scaling ${\rm{PMF}}(\nu )\sim {\nu }^{-3/2}$ which we can call the ‘knot spectrum’ of turbulence.

## Discussion

We have exploited the key property of quantum fluids - the discrete nature of vorticity - to quantify the topology of a small region of quantum turbulence (a vortex tangle) away from boundaries in a statistical steady-state regime. Such inhomogeneous vortex configuration can be realized in experiments in which turbulence is driven by small oscillating objects^22–26^ or by focussing ultrasound^27,28^. The temperature regime which we have chosen is typical of many ^4^He experiments.

We have found that the probability that a vortex loop is knotted increases with the loop’s length as for random knots studied in the context of DNA and macromolecules^8^, and saturates above a characteristic length as for tumbled strings^39^, despite the very different physical mechanisms of agitation of these systems (respectively Brownian motion, mechanical chaos, the Biot-Savart law of Eulerian fluid dynamics). We have also found that, at any instant of time, the vortex tangle contains a distribution of vortex knots. We have quantified the topological complexity of these knots in terms of the degree of the Alexander polynomial which we numerically associate to each vortex loop. Surprisingly, the turbulence always contains some very long vortex loops of great topological complexity, and the distribution of this complexity (measured by the degree of the Alexander polynomials) displays a scaling law, or knot spectrum.

With more computing power available, future work will consider larger, denser vortex tangles at different temperatures and drives, including the zero temperature limit, with the aim of determining the dynamical origin of the characteristic length for the knotting probability and of verifying if the knot spectrum scaling is universal or not. One should also extend this initial study to flows in larger periodic domains (where the turbulence can be driven to a more accurate classical Kolmogorov regime), and to flows within channel boundaries, where tools from braid theory may be used. Finally, it would be interesting to perform a similar analysis using HOMFLYPT polynomials.

## Methods

### Superfluid

We use the Vortex Filament Model (VFM) of Schwarz^40^. The method is based on the observation that the average separation between vortex lines in typical superfluid helium (^4^He) experiments is $\ell \approx {10}^{-5}$ or ${10}^{-6}\,{\rm{m}}$, which is many orders of magnitude bigger than the radius of the vortex core, ${a}_{0}\approx {10}^{-10}\,{\rm{m}}$. We can therefore model superfluid vortex lines as closed space-curves $s(\xi ,t)$ of thickness which is infinitesimal to any other length scale of the flow, where *t* is time and $\xi$ is arc-length. The velocity of a vortex line at the point *s* is given by Schwarz’s equation^40^

1 $$\frac{d{\bf{s}}}{dt}={{\bf{v}}}_{self}+\alpha {\bf{s}}^{\prime} \times ({{\bf{v}}}_{n}-{{\bf{v}}}_{self})-\alpha ^{\prime} {\bf{s}}^{\prime} \times [{\bf{s}}^{\prime} \times ({{\bf{v}}}_{n}-{{\bf{v}}}_{self})],$$

where ${\bf{s}}{\rm{^{\prime} }}=d{\bf{s}}/d\xi$ is the unit tangent vector to the curve at the point **s**, **v**_*n*_ is the velocity of the normal fluid at **s**, and *α* and *α*′ are small dimensionless temperature-dependent friction coefficients^41,42^. The self-induced velocity of the vortex line at the point **s** is given by the Biot-Savart law^43^

2 $${{\bf{v}}}_{self}({\bf{s}})=-\,\frac{\kappa }{4\pi }\,{\oint }_{ {\mathcal L} }\,\frac{({\bf{s}}-{\bf{r}})\times {\bf{d}}{\bf{r}}}{{|{\bf{s}}-{\bf{r}}|}^{3}},$$

where the line integral extends over all vortex lines: ${\mathcal L} ={\cup }_{i=1}^{N}\,{ {\mathcal L} }_{i}$. In the low-temperature limit $T\to 0$, the friction coefficients vanish, vortex lines are simply advected by the flow which they generate, and Schwarz’s equation reduces to $d{\bf{s}}/dt={{\bf{v}}}_{self}({\bf{s}})$ in agreement with the classical Helmholtz’s Theorem. In practice, the zero-temperature limit is a good approximation for for $T < 1\,{\rm{K}}$; At higher temperatures, the normal fluid fraction is not negligible and the friction terms must be included in Schwarz’s equation. In our numerical simulations we choose the temperature $T=1.9\,{\rm{K}}$ which is typical of many experiments and corresponds to $\alpha =0.206$ and $\alpha ^{\prime} =0.00834$; at this temperature, the superfluid and normal fluid fractions are respectively ${\rho }_{s}/\rho =0.58$ and ${\rho }_{n}/\rho =0.42$, where ${\rho }_{s}$ is the superfluid density, ${\rho }_{n}$ the normal fluid density and $\rho ={\rho }_{s}+{\rho }_{n}$ the total density of liquid helium.

Our numerical model uses a variable Lagrangian discretization along the vortex lines^40^ in which the density of discretization points depends on the local curvature. The Biot-Savart integral in equation (2) is de-singularised in a standard way^40^ based on the vortex core cutoff *a*_0_, and the procedure for vortex reconnections is implemented algorithmically^40,44^. The initial condition consists of 40 randomly oriented loops of radius varying according to a normal distribution and with an average number of 200 discretization points on each; the initial rings are located at the centre of the infinite computational domain. The vortex configuration remains localised in a finite volume due to our choice of normal fluid (see below) and temperature, it is important to emphasise that the superfluid velocity is computed in unbounded space, i.e. without the presence of external boundary conditions. The total length of the vortex configuration is

3 $${\rm{\Lambda }}={\oint }_{ {\mathcal L} }\,d\xi ,$$

and similar integrals over distinct loops (${ {\mathcal L} }_{i}$) determine the length of the *i*^th^ loop, ${{\rm{\Lambda }}}_{i}$. Finally, the superfluid energy spectrum *E*(*k*) is defined by

4 $$E=\frac{1}{V}\,{\int }_{V}\,\frac{{\rho }_{s}}{2}|{{\bf{v}}}_{s}{|}^{2}dV={\int }_{0}^{\infty }\,E(k)dk.$$

### Normal fluid

Following the classical turbulence literature, quantum turbulence is usually studied in statistically stationary, homogeneous regimes in three-dimensional periodic domains^45^. However, periodic boundary conditions complicate the definition of the topological properties of the vortex tangle. Fortunately there exist experimental techniques to generate turbulence away from boundaries in ^4^He or ^3^He-B by oscillating objects^22^ such as forks^23^, grids^24,26^, wires^26^, or by focussing ultrasound^27,28^. The microscopic details of the vortex nucleation (e.g. the implosion of cavitating bubbles which create vortex rings^46^ or the interaction with the unavoidable microscopic irregularities of a hard boundary^47^) are not relevant here - we are only concerned on what happens to the vortex lines once they are injected into the fluid.

At finite temperatures, a steady state of quantum turbulence can be maintained by the injection of energy from the normal fluid, whose velocity field we denote **v**_*n*_. Hence, to study the topology of a vortex tangle in a statistically steady state of turbulence, but confined to a finite volume of space, we can make use of a confined normal fluid flow. In a previous study^30^, we made use of random waves modulated by an exponential window so that the velocity field decayed rapidly like ${e}^{-|{\bf{x}}{|}^{2}}$. Whilst this is mathematically convenient, physically it is unrealistic as the normal fluid’s velocity field is no longer solenoidal. Here we make use of a Dudley-James flow^29^, which is well known in the context of dynamo theory and provides a convenient analytic velocity field which is both confined and solenoidal. Importantly, the flow is sufficiently complex to support a magnetic dynamo, and here we shall show it can act effectively as a quantised vorticity dynamo. Using radial coordinates $(r,\theta ,\varphi )$, we assume the following form for the velocity field:

5 $${{\bf{v}}}_{n}(r,\theta ,\varphi )=\sum _{l,m}\,{{\bf{t}}}_{l}^{m}+{{\bf{s}}}_{l}^{m},$$

where,

6 $${{\bf{t}}}_{l}^{m}=\nabla \times \hat{{\bf{r}}}{t}_{l}^{m}{Y}_{l}^{m}(\theta ,\varphi ),\,{{\bf{s}}}_{l}^{m}=\nabla \times \nabla \times \hat{{\bf{r}}}{s}_{l}^{m}{Y}_{l}^{m}(\theta ,\varphi ),\,-\,l\le m\le l.$$

Here we consider the following Dudley & James flow:

7 $${{\bf{v}}}_{n}={{\bf{t}}}_{2}^{0}+\varepsilon {{\bf{s}}}_{2}^{0},$$

with

8 $${t}_{2}^{0}={s}_{2}^{0}={r}^{2}\,\sin (\pi r/D).$$

To introduce time dependence, we take for the normal fluid velocity the form

9 $${{\bf{v}}}_{n}=\{\begin{array}{ll}A(t){{\bf{t}}}_{2}^{0}+B(t)\varepsilon {{\bf{s}}}_{2}^{0}, & r\le D\\ 0, & r > D,\end{array}$$

with $A(t)={v}_{f}\,\sin \,\omega t$ and $B(t)={v}_{f}\,\cos \,\omega t$; in all simulations presented we take $\varepsilon =0.2$, and $\omega =20\,{{\rm{s}}}^{-1}$. We consider two levels of ‘drive’: ${v}_{f}=4.75\,{\rm{cm}}/{\rm{s}}$ and ${v}_{f}=5.25\,{\rm{cm}}/{\rm{s}}$; the value of *v*_*f*_ sets the amount of energy injected from the normal fluid into the superfluid vortex lines, hence the vortex line length in the steady-state regime after the initial transient.

### Alexander polynomial

In order to quantify the topological complexity of the vortex loop ${ {\mathcal L} }_{j}$ we determine its Alexander polynomial

10 $${{\rm{\Delta }}}_{j}(\tau )={a}_{0}+{a}_{1}\tau +\cdots +{a}_{{\nu }_{j}}{\tau }^{{\nu }_{j}}.$$

The degree ${\nu }_{j}$ of the polynomial quantifies the complexity of the loop. For example, the Alexander polynomial of the trivial *unknot* is ${\rm{\Delta }}(\tau )=1$ hence its degree is $\nu =0$. The simplest nontrivial knot is the *trefoil* (3_1_) knot, which has Alexander polynomial ${\rm{\Delta }}(\tau )=1-\tau +{\tau }^{2}$, hence its degree is $\nu =2$. Any vortex loop which has an Alexander polynomial of degree $\nu  > 0$ is knotted (however the converse is not necessarily true: a long-standing problem of knot theory is the lack of a unique method of distinguishing knots from each other; in particular, the Alexander polynomial is not unique to a particular knot type. For example there exist knots which have the same Alexander polynomial as the *unknot*^48^, so the fact that a vortex loop has an Alexander polynomial of degree $\nu =0$ does not necessarily imply that it is an *unknot*).

**Figure 10:**
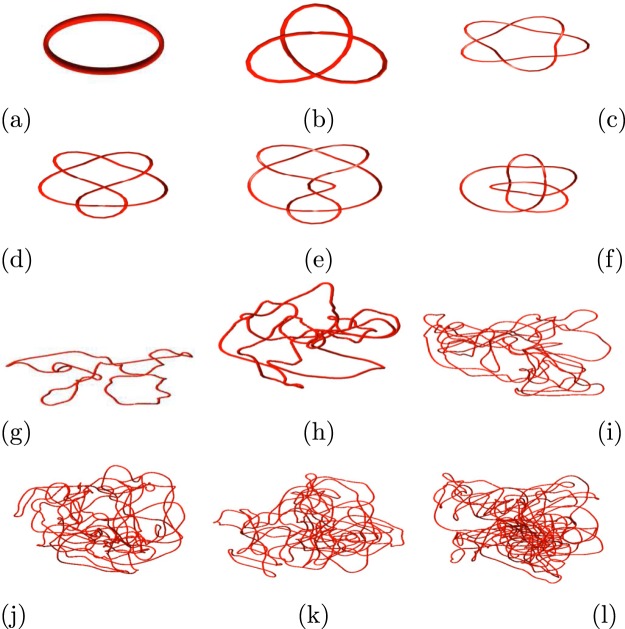
Examples of knots and the degrees of their Alexander polynomials. First (top) row, from left to right: $\nu =0$, $\nu =2$ and $\nu =4$; Second row, from left to right: $\nu =4$, $\nu =4$ and $\nu =4$; Third row, from left to right: $\nu =0$, $\nu =8$ and $\nu =46$; Fourth (bottom) row, from left to right: $\nu =82$, $\nu =108$ and $\nu =232$.

Figure 10 shows a selection of standard and numerically simulated knots with the degree $\nu$ of their respective Alexander polynomials. The six knots on the first two rows are standard knots; the *unknot*, the 3_1_ knot (also known as the trefoil knot), the 5_1_ knot (Solomon’s Seal), the, 6_2_ knot, the 7_5_ knot and the 8_21_ knot. It is apparent that the degree $\nu$ increases with the knots’ complexity. The first (left) knot of the third row, despite its complex appearance, has Alexander polynomial with degree $\nu =0$, and indeed, by untwisting it in visible locations, it can be easily manipulated into an unknot. The remaining knots are obtained from numerical simulations of vortex lines.

Following Livingstone^49^, we compute the Alexander polynomial of a vortex loop by labelling segments of a loop between under-crossings when projected into a plane, followed by assigning coefficients to the relevant entries of a matrix for each segment, and then finding the determinant of the matrix with any single row and column removed^49^. The numerical algorithm, described in R.G. Cooper’s MMath thesis (https://www.jqc.org.uk/publications/theses/), was tested against all the knots of the Rolfsen knot table (http://katlas.org/wiki/The_Rolfsen_Knot_Table). To test knots with very large number of crossings we applied rotations to numerically simulated knots: a rotation changes the number of crossings onto a 2D plane, hence the matrix to determine the Alexander polynomial. A third test consisted in numerically deforming part of a loop adding ‘false’ crossings (which could be untwisted easily if one had the knot in one’s hands): these ‘false’ crossings change the size of the matrix used to compute the Alexander polynomial.

## Data Availability

The datasets generated and analyzed during the current study are available from Newcastle University’s repository.
